# Exploring the antibacterial and dermatitis-mitigating properties of chicken egg white-synthesized zinc oxide nano whiskers

**DOI:** 10.3389/fcimb.2023.1295593

**Published:** 2023-11-28

**Authors:** Sidikov Akmal Abdikakharovich, Mohd A. Rauf, Saadullah Khattak, Junaid Ali Shah, Lamya Ahmed Al-Keridis, Nawaf Alshammari, Mohd Saeed, Sadykov Aslan Igorevich

**Affiliations:** ^1^ Department of Dermatology, Ferghana Medical Institute of Public Health, Ferghana, Uzbekistan; ^2^ School of Life Sciences, Henan University, Kaifeng, Henan, China; ^3^ Miller School of Medicine, University of Miami, Miami, FL, United States; ^4^ College of Life Sciences, Jilin University, Changchun, China; ^5^ Biology Department, Faculty of Science, Princess Nourah Bint Abdulrahman University, Riyadh, Saudi Arabia; ^6^ Department of Biology, College of Sciences, University of Hail, Hail, Saudi Arabia

**Keywords:** ZnO-NWs, antibacterial, anti-biofilm, electron microscopy, *E.coli*, *S.aureus*, skin infection

## Abstract

**Introduction:**

Zinc oxide nanoparticles (ZnO-NPs) have garnered considerable interest in biomedical research primarily owing to their prospective therapeutic implications in combatting pathogenic diseases and microbial infections. The primary objective of this study was to examine the biosynthesis of zinc oxide nanowhiskers (ZnO-NWs) using chicken egg white (albumin) as a bio-template. Furthermore, this study aimed to explore the potential biomedical applications of ZnO NWs in the context of infectious diseases.

**Methods:**

The NWs synthesized through biological processes were observed using electron microscopy, which allowed for detailed examination of their characteristics. The results of these investigations indicated that the NWs exhibited a size distribution ranging from approximately 10 to 100 nm. Fourier-transform infrared spectroscopy (FTIR) and scanning electron microscopy-energy dispersive X-ray spectroscopy (SEM-EDX) mapping analyses successfully corroborated the size, dimensions, and presence of biological constituents during their formation. In this study, XTT assay and confocal imaging were employed to provide evidence of the efficacy of ZnO-NWs in the eradication of bacterial biofilms. The target bacterial strains were Staphylococcus aureus and Escherichia coli. Furthermore, we sought to address pertinent concerns regarding the biocompatibility of the ZnO-NWs. This was achieved through comprehensive evaluation of the absence of cytotoxicity in normal HEK-293T and erythrocytes.

**Results:**

The findings of this investigation unequivocally confirmed the biocompatibility of the ZnO-NWs. The biosynthesized ZnO-NWs demonstrated a noteworthy capacity to mitigate the dermatitis-induced consequences induced by Staphylococcus aureus in murine models after a therapeutic intervention lasting for one week.

**Discussion:**

This study presents a comprehensive examination of the biosynthesis of zinc oxide nanowhiskers (ZnO-NWs) derived from chicken egg whites. These findings highlight the considerable potential of biosynthesized ZnO-NWs as a viable option for the development of therapeutic agents targeting infectious diseases. The antibacterial efficacy of ZnO-NWs against both susceptible and antibiotic-resistant bacterial strains, as well as their ability to eradicate biofilms, suggests their promising role in combating infectious diseases. Furthermore, the confirmed biocompatibility of ZnO-NWs opens avenues for their safe use in biomedical applications. Overall, this research underscores the therapeutic promise of ZnO-NWs and their potential significance in future biomedical advancements.

## Introduction

The presence of pathogens and biofilm formation pose considerable obstacles in the context of wound healing within the broader framework of the global healthcare system (Zhao et al., 2017). Presently, it is estimated that a considerable population of approximately 125 million individuals worldwide is affected by infectious skin diseases (Basra and Shahrukh, 2009; Pezzolo and Naldi, 2020). Skin infections frequently exhibit notable manifestations in infected cutaneous regions, aberrant differentiation of the epidermis, and formation of purulent exudates. The resolution of such health issues necessitates the use of efficacious antimicrobial nano formulations for the treatment or prevention of aa infections. By virtue of their distinct dimensions and configurations, nanomaterials manifest unparalleled attributes in the realms of physics, chemistry, and biology, thereby establishing themselves as pioneering frameworks replete with multifaceted utilities in physical sciences and biomedical disciplines (Peltzer et al., 2009; Gupta et al., 2013; Tambo et al., 2015).

Nanomedicine exhibits considerable potential, not only limited to polymeric carrier-mediated drug delivery platforms but also encompasses biogenic metals and inorganic nanomaterials (Fariq et al., 2017; Rauf et al., 2017; Ahmed et al., 2020). Zinc oxide nanoparticles (ZnONPs) have attracted significant attention as prominent contenders in the field of nanomedicine. When topically administered, zinc oxide (ZnO) nanoparticles have been shown to mitigate allergen-induced skin inflammation (Ilves et al., 2014; Mishra et al., 2017). Experimental amalgamation of ZnO nanorods and diallyl sulfide has exhibited a notable and advantageous synergistic effect on the manifestation and progression of acute dermatitis in animal models, as evidenced by previous research (RAUF et al., 2018). Biogenic zinc oxide (ZnO) nanomaterials coated with bacterial polysaccharides and gelatin biopolymers have been used in a multitude of applications (Basumatary et al., 2022; Priyadarshi et al., 2022). These applications encompass a wide range of activities, including antibacterial, antiviral, antibiofilm, antilarvicidal, and antiangiogenic properties (Wang, 2004; Jones et al., 2008; Dwivedi et al., 2014).

Zinc oxide (ZnO) nanoparticles (NPs) have a diverse array of applications in the cosmetic industry, particularly in the formulation of sunburn lotions. These nanoparticles have garnered recognition from the Food and Drug Administration (FDA) owing to their inherent biocompatibility (Sharma et al., 2012; Mandal et al., 2022). Zinc metal ions are of utmost significance in biological systems, as they function as essential co-factors within select enzymes and actively contribute to a myriad of crucial cellular processes. Previous studies have reported the potential effects of ZnO on the host defense system, cancer prevention, and generation of reactive oxygen species (ROS) in cancer cells have been reported in previous studies (Xie et al., 2011; Akhtar et al., 2012; Sharma et al., 2012). The interaction between ZnO nanomaterials and bacterial membrane lipids has been observed to results in the generation of reactive oxygen species (ROS), which subsequently induce membrane lysis and disrupt cellular integrity. Notably, nanostructures derived from zinc oxide (ZnO) have demonstrated a notable characteristic of inducing minimal toxicity in healthy cells. Furthermore, there have been reports indicating the potential of nanoscale ZnO particles to enhance the functionality of osteoblasts (Nair et al., 2009; Rasmussen et al., 2010; Ye and Shi, 2018).

Conventional methodologies employed for the synthesis of zinc oxide nanoparticles (ZnO-NPs) typically involve the utilization of hazardous substances and require substantial energy input, thereby necessitating the implementation of specific reaction conditions. The presence of toxic chemical conjugants on the surfaces of chemically synthesized nanomaterials can hinder their effectiveness in biomedical applications and raise concerns regarding their potential to induce carcinogenic, genotoxic, and cytotoxic effects. Consequently, an escalating demand has emerged for the development of more secure and ecologically sustainable approaches for the synthesis of nanoscale materials intended for biomedical applications. The process of nanoparticle biosynthesis presents numerous advantageous attributes, including temporal and energetic efficiency, as well as environmental compatibility. The use of various biological entities such as plant extracts, bacteria, fungi, and mammalian cells has been extensively investigated in the context of nanoparticle synthesis. However, the potential of egg white protein as a biosynthetic agent for nanoparticle fabrication has received limited attention, thereby presenting a novel and environmentally sustainable avenue for exploration (Hernández-Sierra et al., 2008; Jones et al., 2008; Xie et al., 2011; Mahamuni-Badiger et al., 2020).

This study employed chicken egg white, specifically albumin protein, as a substrate for the biosynthesis of nontoxic and biocompatible ZnO nanowhiskers (Khan et al., 2011; Khan et al., 2015; RAUF et al., 2018). The present study delved deeper into the examination of the biomedical applications of the nanowhiskers under investigation, specifically in their capacity as antibacterial agents targeting both gram-negative *Escherichia coli* and gram-positive *Staphylococcus aureus*. These bacterial strains are widely recognized as significant contributors to a diverse range of infections. The investigation included both *in vitro* and *in vivo* experiments, thereby encompassing controlled laboratory conditions as well as real-life biological systems. The present findings have the potential to engender novel pathways for the advancement of efficacious and secure antimicrobial approaches for the management of infectious ailments.

## Materials and methods

All chemicals and reagents used in this study were of high quality and were procured from Sigma-Aldrich. Inbred BALB/c mice (6–8 weeks old, 20 ± 2 g) were obtained from Henan Animal House Facility. All mice were housed in polypropylene cages and maintained under optimum temperature conditions on a 12 h light-dark cycle. All animals were provided pallet feed and water ad libitum in an environmentally controlled house. All animal handling protocols and experiments were approved by the Medical and Scientific Research Ethics Committee of Henan University School of Medicine (P. R. China).

### Biofabrication of ZnO nanowhiskers from chicken egg white

ZnO-NWs were fabricated from hen egg white, using our previously published article (Naqvi et al., 2013). Briefly, eggs were cleaned and sanitized using 75% ethanol to ensure the absence of mycoplasma infection by testing each egg content. The eggshell was then broken, and the egg white was separated from the yellow yolk part and stored at 4°C. Initially, a mixture of 15 mL of deionized water and 30 mL of egg white was vigorously stirred at an ambient temperature of 25°C. Stirring was continued until a uniform solution was obtained, indicating the homogeneity of the mixture. A homogeneous mixture was obtained by gradually introducing a 0.1 N solution of zinc acetate into the egg white solution under vigorous stirring at a temperature of 25°C over a period of four hours. Following the initial step, 20 mL of the solution was carefully transferred to a microwave glass tube with a capacity of 30 mL, which was securely sealed using a septum. The experimental procedure involved subjecting the samples to microwave irradiation using an Anton Paar Monowave 300 instrument. The reaction was carried out at 200°C for 60 min. Subsequently, the sample was cooled in a microwave reactor until it reached 40°C, resulting in the formation of a solution containing a milky white precipitate. Later, the resultant precipitate, which was synthesized through a series of experimental procedures, underwent centrifugation, followed by multiple washes with water. Next, vacuum drying was performed at 80°C for 6 h, ultimately yielding the ZnO-NWs.

### Characterization of synthesized ZnO-NWs

The synthesized ZnO-NWs were characterized using various advanced techniques, such as UV-visible spectroscopy, FTIR and electron microscopy. Information regarding the detailed methodology used is provided in the **Supplementary Material**.

### Bacterial strains and cell culture conditions

In this study, we utilized two distinct bacterial species as focal test organisms: *Escherichia coli*, a gram-negative bacterium, and *Staphylococcus aureus*, a gram-positive bacterium. These microorganisms were selected for the majority of the experimental investigations conducted throughout the course of this research endeavor. The Luria Bertani (LB) broth and nutrient agar as culture media was used to facilitate the cultivation of the bacteria. Experiments were conducted at 37°C in a rotary orbital shaking incubator. The optical density of the bacterial culture was measured at a wavelength of 600 nm.

### Determination of minimum inhibitory concentration and antibacterial efficacy through agar diffusion assay

Determination of the Minimum Inhibitory Concentration (MIC) is a crucial parameter in assessing the sensitivity of microorganisms to a particular drug or material. For ZnO-NWs, the MIC was determined against standard bacterial strains and clinical isolates, including *Staphylococcus aureus, Enterococcus faecalis*, *Pseudomonas aeruginosa*, MRSA, *Listeria monocytogenes*, and *Escherichia coli.*

The MIC value of the ZnO-NWs was determined using a 96-well plate microdilution method. This method involved preparing a series of dilutions of ZnO-NWs in a 96-well plate, with each well containing a different concentration of the nanomaterial. The bacterial strains were inoculated into the wells and the plate was incubated for a specified period of time.

After incubation, the MIC was determined as the lowest concentration of ZnO-NWs that completely inhibited visible growth of the microorganism within the specified time frame. This provides valuable information on the effectiveness of ZnO-NWs in inhibiting the growth of different bacterial strains, and the antibacterial efficacy of ZnO-NWs was also evaluated through an agar diffusion assay. In this assay, agar plates were prepared with a bacterial lawn, and wells were made in the agar with the gel puncture and sealed with 1% agarose. ZnO-NWs were then poured to the wells, and then, plates were incubated. The antibacterial activity of the ZnO-NWs was assessed by measuring the zone of inhibition around the wells, which indicates the area where bacterial growth was inhibited by the ZnO-NWs. The detailed methodology and results of both MIC determination and agar diffusion assays are provided in the **Supplementary Section** of the study, which provides comprehensive insights into the antibacterial potential of ZnO-NWs against different bacterial strains, including both standard laboratory strains and clinical isolates.

### The antibiofilm potential of ZnO-NWs

The antibiofilm potential of ZnO-NWs was assessed using XTT-based biofilm testing following a previously published method (Xu et al., 2016). The XTT assay is a colorimetric method used to measure the metabolic activity of biofilms and provides insight into their viability and susceptibility to antimicrobial agents.

Biofilm formation was initiated in the wells of a plate, and after the formation of mature biofilms, nonadherent cells were removed by washing with sterile PBS. Selected wells with mature biofilms were treated with varying concentrations of ZnO-NWs for 48 h. The detailed methodology for preparing the samples for the XTT assay is provided in the **Supplementary Section**.

In addition to the XTT assay, crystal violet staining was performed to assess biofilm formation. After h 48-hour incubation with the ZnO-NWs, the wells were washed with PBS and dried in an inverted position on paper towels. Biofilm formation was quantified by staining the biofilms with 0.1% crystal violet solution and incubating them for 15 min at room temperature. The excess stain was washed with deionized water and the wells were dried thoroughly (Qayyum et al., 2017; Oves et al., 2022).

The combination of the XTT assay and crystal violet staining, helped incomprehensive evaluation of the antibiofilm activity of ZnO-NWs. The XTT assay provides information on the metabolic activity and viability of biofilms, whereas crystal violet staining quantifies biofilm formation. Taken together, these assays offer valuable insights into the ability of ZnO-NWs to inhibit and disrupt biofilm formation, which is crucial for combating bacterial infections and preventing their recurrence.

### Nanoparticle treated bacterial biofilm and hydrophobicity index

Both treated and controlled overnight grown bacterial cultures were centrifuged at 5000 rpm for 10 min at 4°C, the obtained pellets were resuspended in BHI broth, and turbidity was maintained according to the McFarland standard. Culture OD up to 1.0 ± 0.01 at 595 nm wavelength.

Subsequently, 0.1 ml toluene was added to the cell suspension in a test tube and vortexed. The biphasic mixture of the two phases was allowed to settle for 30 min and the optical density of the aqueous phase was measured. The hydrophobicity index (HI) of the aqueous phase was then obtained from the microbial cells. The hydrophobicity of the aqueous phase obtained from microbial culture was analyzed by using the following equation-


$$\%HI=\left[\left(A_{i}-IA_{f}\right)/A_{i}\right]\times 100$$

Where A_i_ and A_f_ are the initial and final optical densities of the aqueous phase obtained from bacterial culture, respectively. The hydrophobicity of the bacterial cells was estimated by evaluating their adherence to organic solvent like toluene (Serebriakova et al., 2002).

### Bacterial susceptibility against as-synthesized ZnO-NWs

CFU count and growth curve assays were performed to determine the *in vitro* efficacy of the synthesized ZnO-NWs. A detailed methodology of the test is provided in the **Supplementary Section**.

### Bacteria -NCs interaction as revealed by electron microscopy

The effect of different formulations of ZnO-NWs on the surface morphology of *S. aureus* and *E. coli* was further studied using different electron microscopy techniques. The detailed methodology of the sample preparation is provided in the **Supplementary Section**.

### Protein leakage assay

For quantitative analysis of protein leakage from the studied bacterial cells, the assay was performed using a previously developed protocol (Pati et al., 2014). Briefly, 30 mL of different strain (*E. coli* and *S. aureus* ∼10^9^ CFU/mL) suspensions in 0.9% NaCl were mixed with ZnO-NWs at sub-MIC concentrations. The control group consisted of untreated cells. After exposure, the samples were incubated for 3 h at 37°C, followed by centrifugation at 10,000 rpm at 4°C for 10 min. The supernatant was immediately harvested and lyophilized, and the protein concentration of each sample was quantified using the BCA assay.

### Intracellular ROS production by biosynthesized ZnO-NWs

ZnO-NWs mediated generation of intracellular ROS in treated bacterial cells were measured by employing fluorescent probe 2,7-dichlorofluorescein diacetate (DCFH-DA) (Eruslanov and Kusmartsev, 2010). Cells were observed under a fluorescence microscope. The detailed methodology is provided in the **Supplementary Data**.

### Bacterial cell viability assay using SYTO and propidium iodide staining

To assess the viability of the bacterial strains, E. coli and S. aureus following treatment with ZnO-NWs were observed using a SYTO9-PI bacterial viability kit (Invitrogen, CA, United States) (Deitch et al., 1982; Ansari et al., 2018). A detailed description is provided in the **Supplementary Information**.

### *In vitro* erythrocyte lysis test

For potential biomedical applications, the cytotoxicity of the biosynthesized ZnO-NWs was studied in RBCs and the HEK-293 cell line. First, an *in vitro* erythrocyte lysis experiment was performed. In this experiment, nanomaterials interacted with erythrocytes, causing membrane leakage and disruption of hemoglobin, and the released hemoglobin was measured. Next, the MTT-based viability of the HEK-293 cells was determined. Detailed information is provided in the **Supplementary Information file**.

### Determination of ZnO-NWs antibacterial potential against murine dermatitis

The antibacterial potential of the ZnO-NWs against murine dermatitis was determined using a mouse model of skin infection caused by S. aureus. The test animals were divided into four groups of five mice each. To induce infection, a ketamine-xylazine cocktail solution was injected intraperitoneally (IP) for anesthesia, and a sterile scalpel blade was used to create a reddened area on the shaved dorsal surface of the mouse skin. The skin surface was then inoculated with a suspension of *S. aureus* (50 μl, 10 (Fariq et al., 2017) colony-forming units).

The following experimental groups were analyzed:

1. Group: Mice infected with *S. aureus.*
2. Group: Mice were exposed to *S. aureus* and treated with ZnO-NWs (0.5 g/kg body weight).
3. Group: Mice treated with ZnO-NWs (1 g/kg body weight).
4. Group: Control group treated with phosphate-buffered saline (PBS) only.

After allowing the infection to establish for five days, ZnO-NW topical treatment was applied for seven days, three times a week. After one week of treatment, the animals were euthanized under general anesthesia with a ketamine-xylazine cocktail solution. Infected skin samples were collected in sterile tubes and homogenized using a tissue homogenizer. The homogenized samples were serially diluted and the residual *S. aureus* burden was determined by plating on BHI plates.

In addition to the bacterial burden assessment, infected skin tissues were subjected to histopathological analysis. Infected tissues were collected from each group, sliced into small pieces, and fixed in a 10% formaldehyde solution. A standard paraffin tissue embedding technique was used to prepare histopathology slides. The slides were stained with hematoxylin and eosin (H&E) stain and examined under an Olympus BX40 microscope. The inflammatory response in tissues was scored based on the presence of inflammatory cells, with scores ranging from 0 (no inflammation) to 3 (severe inflammation). This study aimed to evaluate the antibacterial effects of ZnO-NWs in the context of murine dermatitis caused by S. aureus infection. Bacterial burden assessment and histopathological analysis provided important insights into the potential therapeutic benefits of ZnO-NWs in treating skin infections, as well as their impact on the inflammatory response in infected tissues.

### *In vivo* animal imaging and LFT (liver function test assays) for biocompatibility

A cohort of 9 female BALB/C mice were randomly assigned to 3 groups: mice injected with FITC-tagged ZnO-NWs (1g/kg body wt) and FITC-tagged ZnO-NWs (0.5g/kg body wt), and control (PBS) group, with each group consisting of 3 mice. The mice used in the study were between 6 and 8 weeks old, and had an average body weight of 18 ± 2 grams. The subjects were administered a 100 μl injection of FITC-tagged ZnO-NWs through the tail vein. The mice were imaged using the small animals *in vivo* bioimaging system (IVIS Lumina III, PerkinElmer) after 24 hours. In addition, the essential organs, such as the liver, spleen were extracted and subjected to imaging for fluorescence intensity, which served as a measure of the accumulation of ZnO-NWs in these key organs. The imaging process involved excitation at a wavelength of 488 nm and emission at 670 nm. In addition, blood were taken out through retro-orbital puncture and subjected to hepatotoxicity assays like ALT, AST and ALB.

### Statistical analysis

Statistical calculations were performed using GraphPad Prism (version 9.0; GraphPad Software Inc., San Diego, California, USA). All results are expressed as mean ± SEM. Data were analyzed using one-way analysis of variance (ANOVA) and two-way ANOVA to assess differences among the various groups. Significance is indicated by ***P ≤0.001, **P ≤ 0.01, and *P ≤0.05. The Student’s T-test was used to measure biochemical differences between treatment groups, and differences were considered significant at P ≤ 0.05.

## Results and discussion

### Synthesis and characterization of synthesized ZnO-NWs

The experimental procedure for the fabrication of ZnO nanowhiskers (ZnO-NWs) involved the use of egg white as a biotemplate. Egg white primarily consists of polypeptide chains comprising hydrophilic functional groups, including -NH2,–O-, -CH2-CH2, and -COOH. The proteins in question function as assembly substrates, facilitating the creation of an optimal setting for the crystallization of Zn(OH)_4_^2-^.

During the synthesis, Zn(OH) _4_^2-^was observed. crystals were initially incorporated into the elongated chain alignment of the egg white matrix. During the course of the reaction, Zn(OH)_4_^2 −^ crystals played a significant role as nucleation sites for subsequent crystal growth. The Zn(OH) _4_^2-.^ crystals underwent progressive transformation, evolving into elongated structures resembling whiskers, which spanned the entirety of the egg-white matrix. The progressive development and organization of the crystalline entities of ZnO nanowhiskers have led to the gradual establishment of their three-dimensional configurations.


$$\text{Egg White }\left(–\text{N}{\text{H}}_{2}\text{from ovalbumin}\right)\;+\;{\text{H}}_{2}\text{O}⟶\text{Egg White }\left(–\text{N}{\text{H}}_{2}^{+}\right)\;+\text{ O}{\text{H}}^{-}$$

$$\text{Z}{\text{n}}^{2+}+\;2\text{O}{\text{H}}^{-}⟶\text{Zn }{\left(\text{OH}\right)}_{2}⟶\text{ZnO }+\;{\text{H}}_{2}\text{O}$$

The synthesized ZnO nanowhiskers (ZnO-NWs) underwent a comprehensive characterization process to gain insight into various aspects of their properties. This includes examination of their size, surface characteristics, elemental composition, optical behavior, functional groups, and crystal structure.

**Figure 1:**
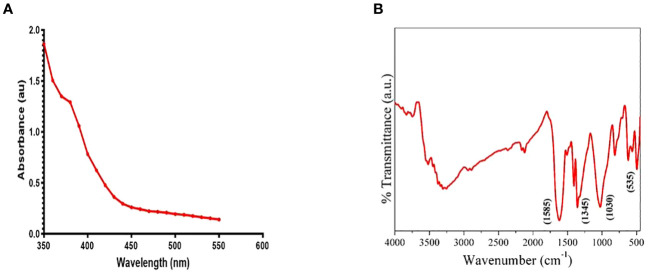
**Figure 1** presents the characterization of the synthesized ZnO-NWs. **(A)** The present study focuses on the UV–vis absorption spectrum analysis of biogenic ZnO-NWs, which were synthesized using an egg white as a biotemplate. **(B)**The Fourier Transform Infrared (FTIR) pattern exhibited by nanoparticles (NPs) serves as a representation of their synthesis process.

The optical properties of the ZnO nanowhiskers (ZnO-NWs) were examined using UV-Vis spectroscopy, which revealed the presence of a discernible absorption peak at a specific wavelength of 370 nm (**Figure 1A**). The functional group characteristics of the ZnO-NWs were identified using Fourier-transform infrared spectroscopy (FTIR), as shown in **Figure 1B**. The analysis revealed a comprehensive and extensive increase within the spectral region spanning 3500–3300 cm−1, which could be attributed to the stretching vibrations of the hydroxyl (OH) groups. Spectral analysis revealed that the prominent peak observed at 1585 cm−1 could be attributed to the bending vibration of the hydroxyl (OH) group. Additionally, the presence of peaks at wavenumbers of 830 cm−1 and 535 cm−1 indicates the occurrence of Zn-O stretching vibrations.

### Analysis of size and surface characteristics

**Figure 2:**
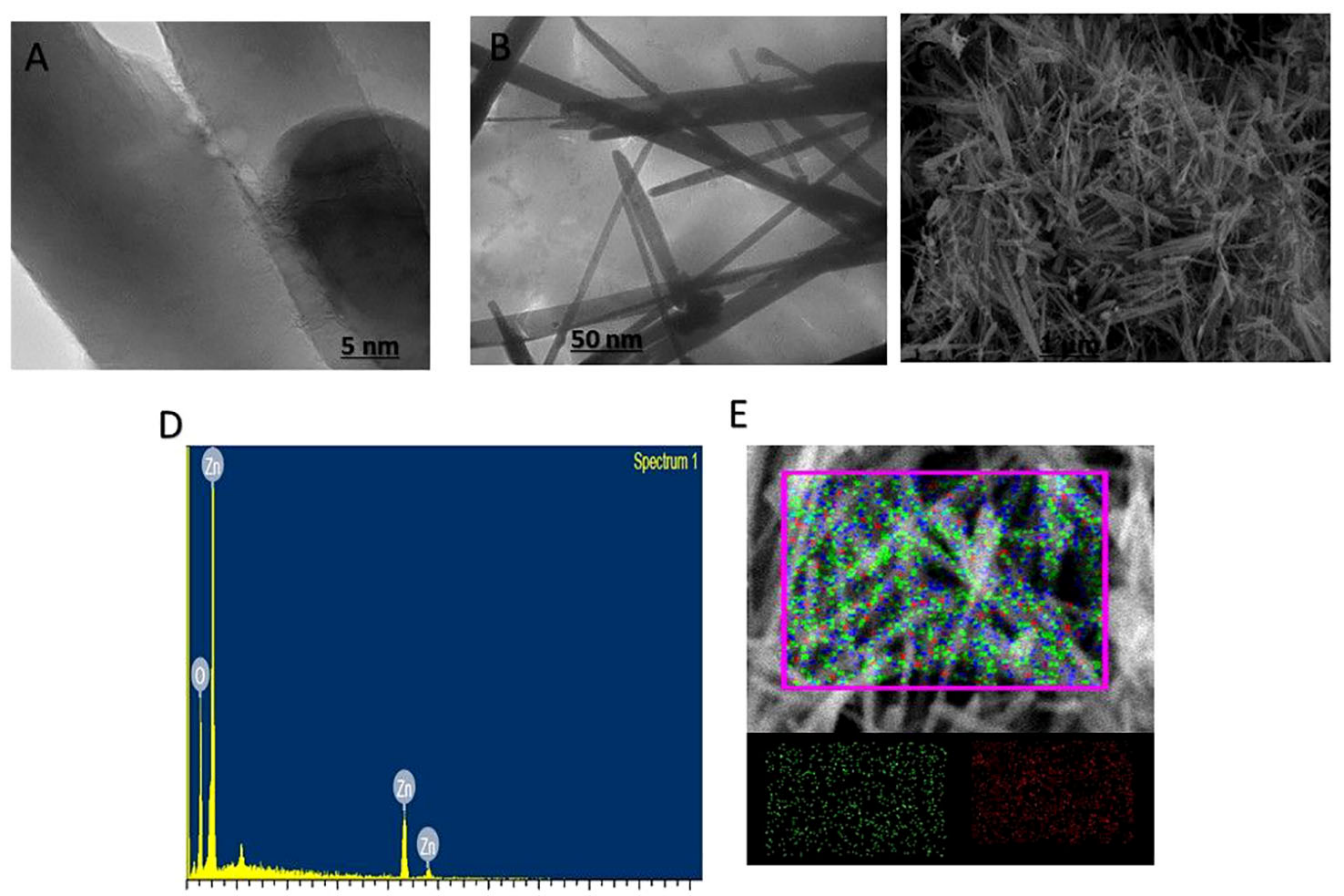
**Figure 2** presents the characterization of the as-synthesized ZnO-NWs. The present study examines the electron microscopy of the synthesized nanoparticles (NPs) denoted as **(A, B)** at different resolutions. **(C)** represents the scanning electron microscopy (SEM) images of ZnO-NMs, which provide insights into the microstructure of the synthesized NPs. **(D, E)** The elemental composition of as-synthesized ZnO-NWs is confirmed by the EDAX spectrum.

The utilization of transmission electron microscopy (TEM) imaging techniques facilitated the examination of the synthesized zinc oxide nanowhiskers (ZnO-NWs), revealing their heterogeneous composition, characterized by the presence of clusters and nanowhiskers with dimensions within the range of 10-50 nm (as depicted in **Figure 2**). The size of the ZnO-NWs was determined to be approximately 40 nm using scanning electron microscopy (SEM), as depicted in **Figure 2C**.

### Elemental analysis

The Energy-dispersive X-ray spectroscopy (EDX) was employed for elemental analysis, which yielded valuable insights into the composition of the synthesized ZnO-NWs. The results obtained from this analysis revealed that the highest concentrations of Zn and oxygen were detected in the ZnO-NWs, as depicted in **Figures 2D, E**. The utilization of EDX mapping techniques effectively corroborated the purity of the material and absence of elemental contamination.

The findings from the characterization analysis provided compelling evidence that the synthesis of ZnO-NWs was indeed successful, as indicated by the well-defined dimensions, structural integrity, and high level of purity. The present findings establish a fundamental basis for the investigation of the biomedical potential of ZnO-NWs, with a specific focus on their efficacy as antibacterial agents that target pathogenic bacteria.

### The antibacterial potential of ZnO-NWs

The antibacterial potential of the biosynthesized ZnO-NWs was evaluated against medically important pathogens. Ampicillin was used as a control for comparison. The Minimum Inhibitory Concentration (MIC) values for the synthesized nanoparticles were determined and are listed in **Table 1**.

**Table 1:** MIC as observed by ZnO–NPs against enlisted tested microbes by 96-well plate microdilution method*^a^*.

Strains	Zinc oxide NPs (in ug/ml)
*a* MIC observed by 96-well plate microdilution method.	
*S. aureus* ATCC 25923	32
MRSA 1	256
*Listeria monocytogenes*	64
*Escherichia coli* ATCC 25922	32
MREC	128
*Enterococcus faecalis*	128
*P. aeruginosa*	64

**Figure 3:**
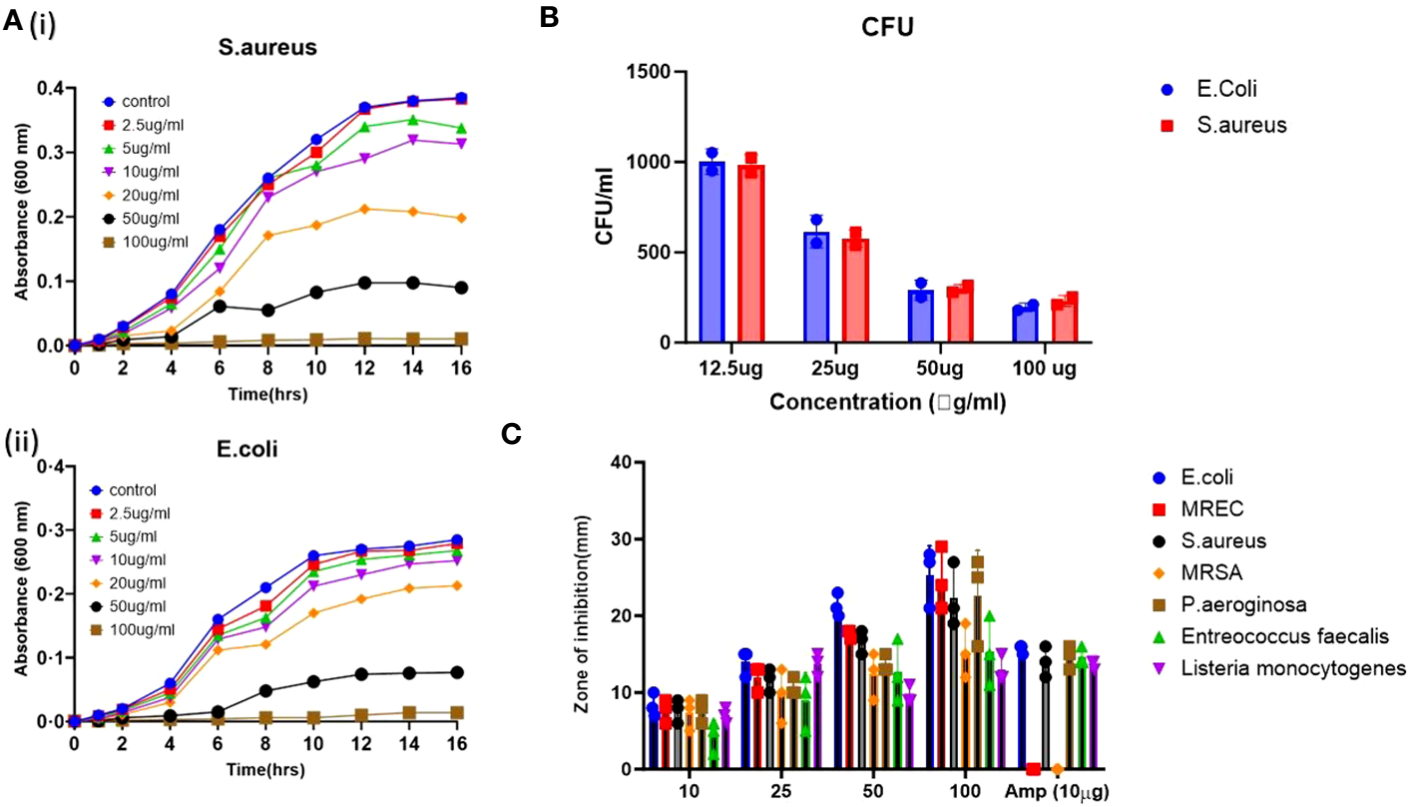
The antibacterial activity of the synthesized ZnO-NWs. The present study investigates the antibacterial activity of biosynthesized ZnO-NWs in two separate experiments, denoted as 3 **(A, B)**. The first experiment focuses on the growth kinetics of Staphylococcus aureus (S. aureus) and Escherichia coli (E. coli) over varying time intervals, while the second experiment examines the growth kinetics of these bacteria at different concentrations of ZnO-NWs. The CFU assay was conducted to evaluate the antimicrobial activity against microorganisms. The Gram-positive bacterium *Staphylococcus aureus*. Furthermore, the second category of bacteria under investigation is Gram-negative *Escherichia coli* (E. coli). **(C)**, The agar diffusion assay was conducted to assess the inhibitory effects of ZnO-NWs on different microorganisms. The resulting bar graphs depict the zones of inhibition induced by the ZnO-NWs.

Additionally, a zone inhibition assay was performed using the agar well diffusion method to assess the bacterial growth inhibition by the applied ZnO-NWs (**Figure 3C**), and growth kinetics were observed while co-culturing the ZnO-NWs with the bacteria, showing a reduction in the growth of bacteria over a period of 24 h with an increase in dosage (**Figure 3A**). The biosynthesized ZnO-NWs had a larger specific surface area, facilitating more efficient contact with the target bacterial cells. Once the ZnO-NWs were attached to the bacterial cell walls, reactive oxygen species (ROS) generation commenced, leading to significant inhibition of bacterial growth. This mechanism is supported by previous studies (Patra et al., 2015; Sirelkhatim et al., 2015; Gold et al., 2018) in which zinc oxide nanoparticles were found to extensively interact with bacterial cell walls, resulting in cell lysis (**Figure 3B**).

The interaction between nanomaterials and the bacterial cell membrane triggers lipid peroxidation initiated by hydroxyl radicals (OH), leading to membrane disintegration and eventual bacterial cell death (Guo et al., 2009; Wang et al., 2017). Moreover, ZnO nanoparticles can physically adhere to the bacterial cell surface, contributing to the killing of the target bacteria, and the results demonstrate the potent antibacterial activity of the biosynthesized ZnO-NWs against medically important pathogens. The ability of these nanowhiskers to interact with bacterial cells and induce ROS generation makes them promising candidates for combating bacterial infections.

### Antibiofilm & hydrophobicity index of as-synthesized ZnO-NWs

**Figure 4:**
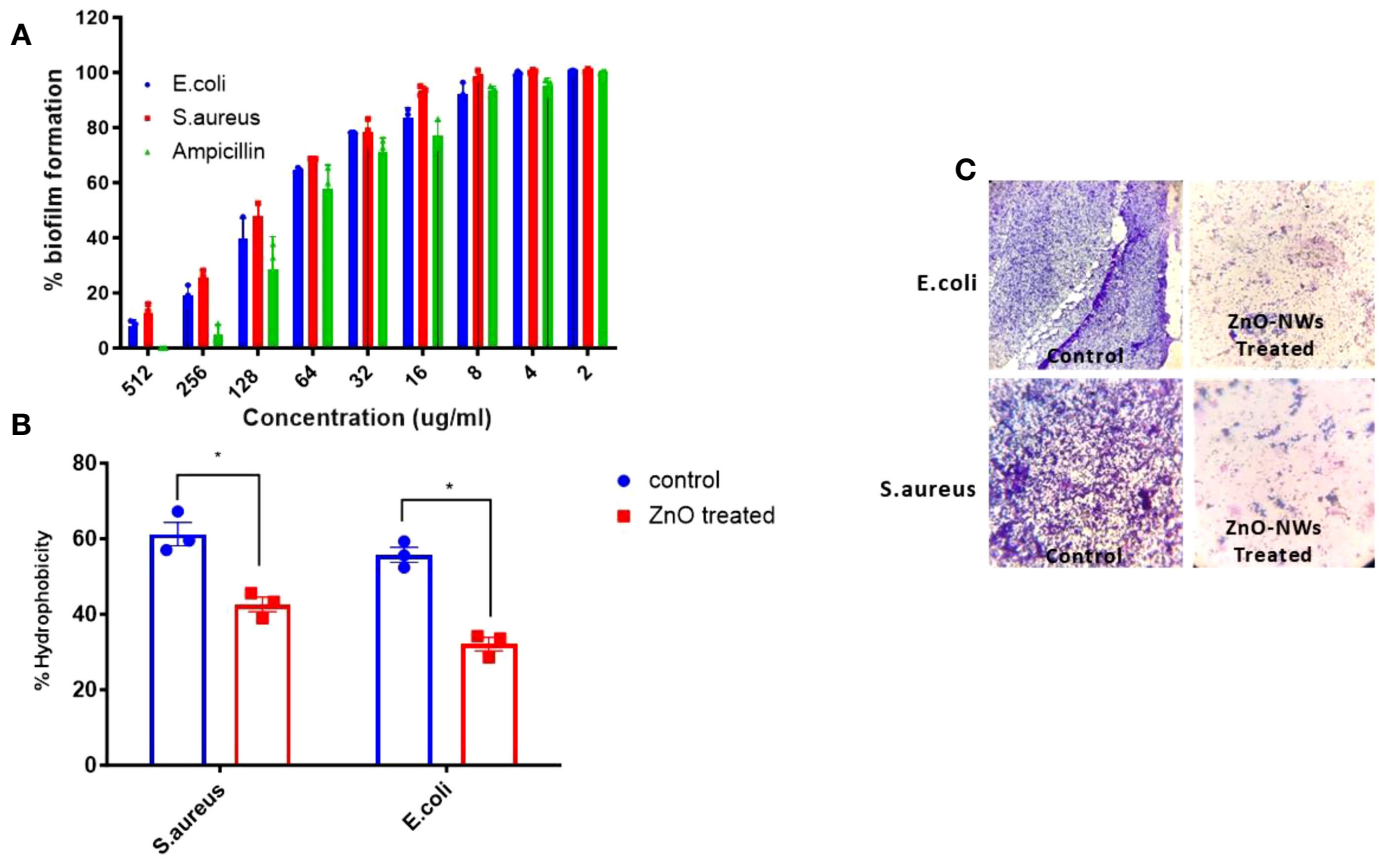
The Effect of ZnO-NMs on inhibition of biofilm development. The present study investigates the impact of Zinc Oxide nanowhiskers (ZnO-NWs) on the inhibition of biofilm formation. The inhibition of biofilm growth was assessed by quantifying the relative metabolic activity (RMA) using the XTT assay, with the untreated control set as the reference point at 100%. **(B)** The hydrophobicity index of both *E.coli* and *S. aureus* was assessed subsequent to their exposure to a concentration of 100 μg/ml for a duration of 48 h. The scanning electron microscopy (SEM) technique was employed to visually demonstrate the biofilm formation of *S.aureus* and *E.coli* in the presence of Zinc Oxide nanowhiskers (ZnO-NWs) **(C)**, the provided crystal violet staining image depicts the biofilms under observation in the presence of Zinc Oxide nanowhiskers (ZnO-NWs).

The antibiofilm potential of the synthesized zinc oxide nanowhiskers (ZnO-NWs) was assessed using an XTT assay. The results revealed a discernible dose-dependent effect on biofilm formation (**Figure 4A**). Biofilms represent intricate assemblages of bacterial populations enveloped within a matrix of extracellular substances synthesized by the bacteria themselves. These biofilms present a formidable obstacle to infection control, necessitating innovative strategies for their eradication. The bacterial cell surface exhibits a notable degree of hydrophobicity, which facilitates its ability to adhere to a diverse range of surfaces encompassing mucosal epithelial cells and phagocytes.

Following the application of ZnO-NWs to bacterial cells, a notable decline in the hydrophobicity index was observed, resulting in a substantial 54% decrease in E. coli and 42% decrease in S. aureus (**Figure 4B**). The observed decrease in hydrophobicity can be interpreted as an indication that treatment with ZnO-NWs perturbed the adhesive properties of bacteria, thereby potentially impeding their capacity to form biofilms. Evidence for the disruption of biofilm formation was further substantiated using crystal violet staining. These methodologies revealed a noticeable decrease in the formation of microcolonies within bacterial cells that had been subjected to treatment with ZnO-NWs (**Figure 4C**). The present study revealed that synthesized zinc oxide nanowhiskers (ZnO-NWs) exhibit noteworthy antibiofilm characteristics, effectively impeding the formation of biofilms, thereby presenting a promising avenue for addressing biofilm-related infections. The efficacy of ZnO-NWs in mitigating bacterial hydrophobicity potentially plays a pivotal role in their efficacy in impeding bacterial adhesion and subsequent biofilm formation.

### ZnO-NWs effect on bacterial cell surface

**Figure 5:**
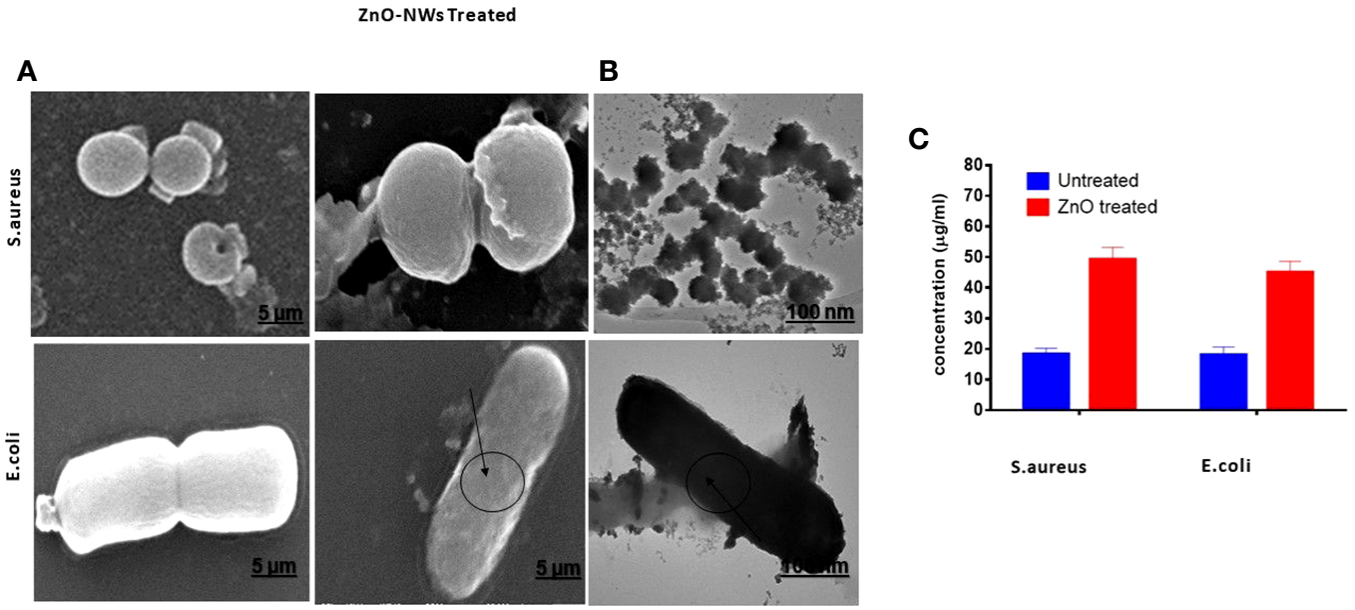
Electron microscopic examination of bacterial morphology after ZnO-NMs exposure. The figure showcases the results of electron microscopic examination conducted to analyze the bacterial morphology subsequent to exposure to ZnO-NWs. The present study investigates the impact of ZnO-NWs on two bacterial strains, namely *S.aureus* and *E.coli.* Specifically, the effects of ZnO-NWs on these bacterial strains are examined separately **(A, B)**. Additionally, the study explores the release of protein from the bacterial membrane subsequent to exposure to ZnO-NWs **(C)**.

The present study aimed to investigate the effect of ZnO-NWs on the surface of bacterial cells using electron microscopy. In the experimental setup involving *E. coli*, the bacterial cells in the control group retained their characteristic rod-shaped morphologies. Furthermore, each individual cell exhibited a relatively consistent size and displayed no discernible indication of surface impairment, as shown in **Figure 5A**. Upon exposure to ZnO-NWs, a notable alteration in the morphological characteristics of the bacterial cells was observed. The treated bacteria displayed anomalous cell surface fragments, which indicated cellular swelling and agglomeration. The aforementioned observations have provided compelling evidence that the presence of ZnO-NWs significantly disrupts the cellular membrane of *Escherichia coli* (E. coli), ultimately resulting in the destruction of bacterial cells. Notably, in the context of *Staphylococcus aureus*, a bacterium known for its characteristic round and smooth morphology, the introduction of zinc oxide nanowhiskers (ZnO-NWs) elicited a sudden and significant alteration in both cellular structure and surface integrity (**Figures 5A, B**).

**Figure 6:**
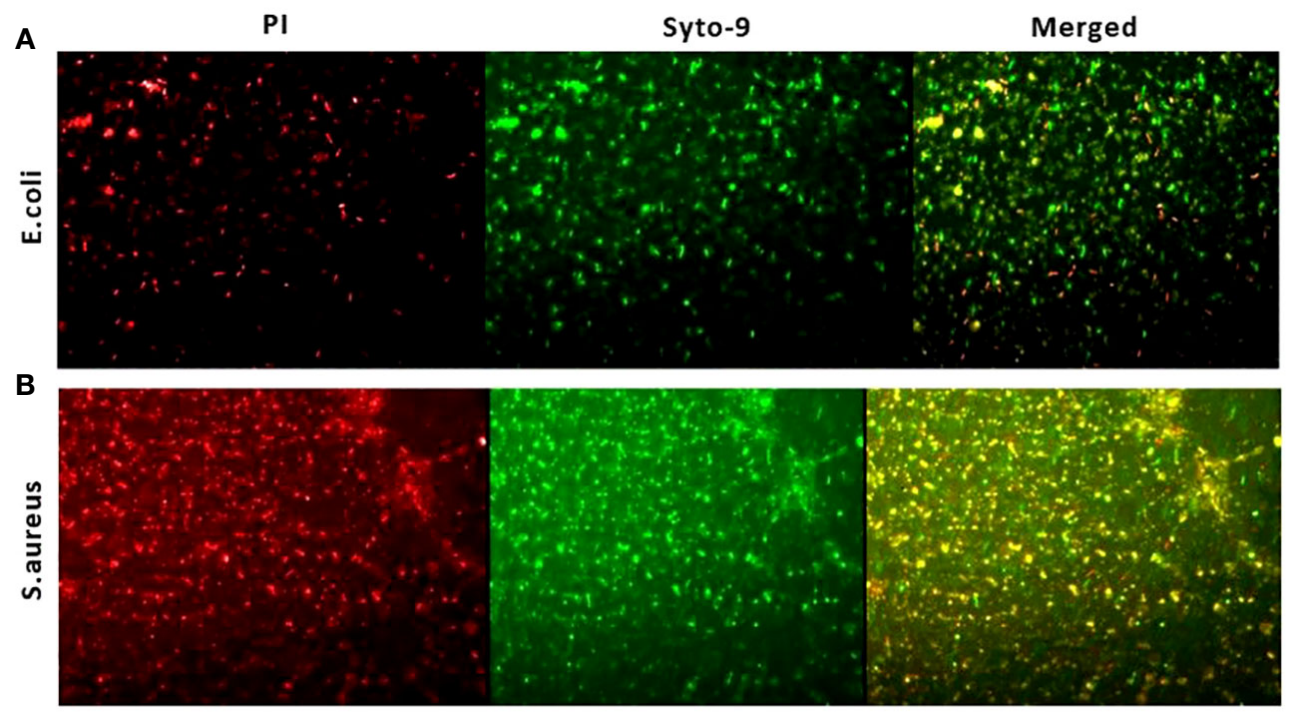
Live dead assay to study antibacterial activity. The fluorescence microscopic images presented in this study depict the results of a live-dead cell assay conducted using propidium iodide (PI) and Syto-9 dyes. The purpose of this assay was to evaluate the anti-bacterial efficacy of zinc oxide nanowhiskers (ZnO-NWs) against E.coli and S.aureus cells.

Further the antibacterial efficacy of the ZnO-NWs synthesized in this study was further validated by confocal microscopy. The application of ZnO-NWs to bacterial cells yielded a notable increase in propidium iodide (PI) staining within the treated cells, thereby signifying a substantial reduction in bacterial viability (**Figure 6**).

The combined utilization of scanning electron microscopy (SEM) and confocal microscopy techniques yields compelling visual documentation that supports the assertion of robust antibacterial efficacy of ZnO nanowhiskers (ZnO-NWs). The empirical examination of morphological alterations and the resultant cell surface impairment implies that the presence of ZnO-NWs disrupts the structural integrity of bacterial cells, ultimately culminating in their death. The present findings provide empirical evidence that ZnO-NWs have considerable potential as efficacious antibacterial agents to mitigate pathogenic bacterial infections.

### Hemolysis assay

**Figure 7:**
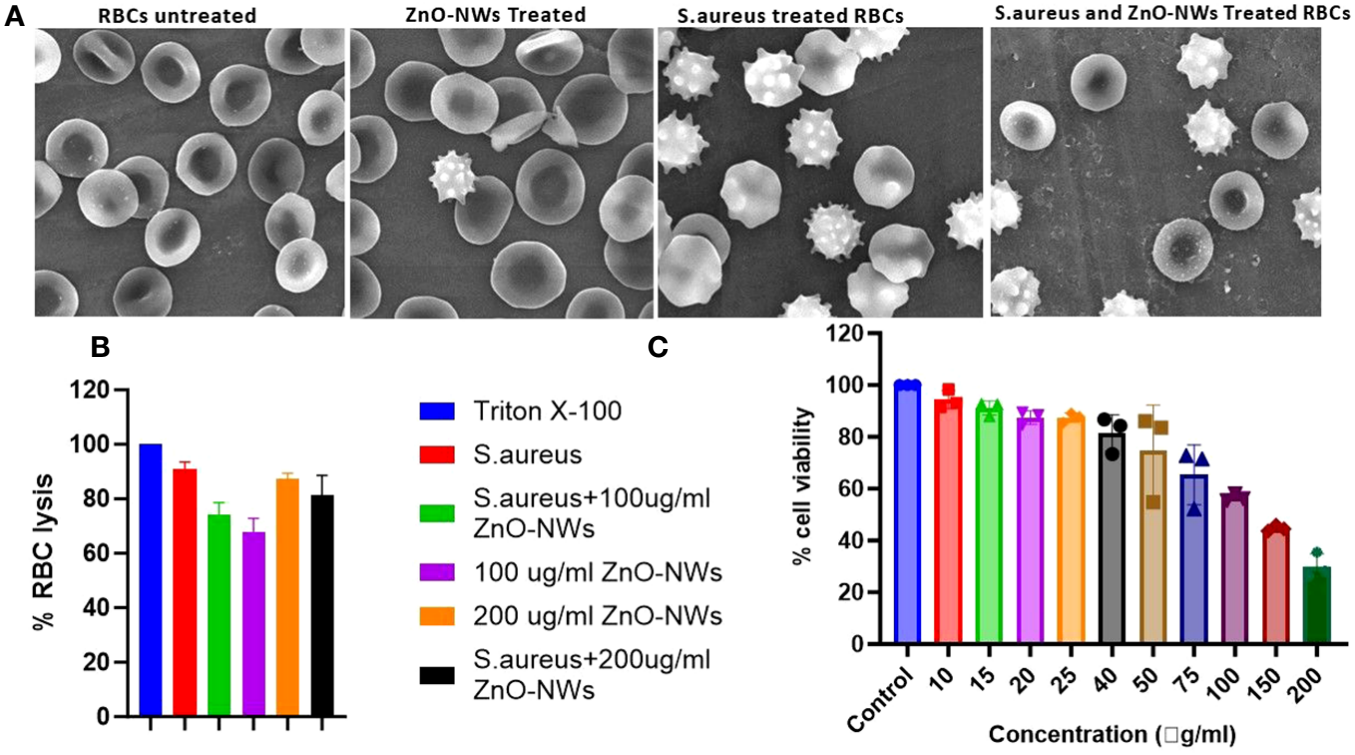
ZnO-NWs inhibited Staphylococcus aureus induced RBC lysis. The study findings indicate that the utilization of ZnO-NWs effectively suppressed the process of red blood cell (RBC) lysis induced by Staphylococcus aureus. The provided SEM micrographs depict the process of red blood cell (RBC) lysis induced by S. aureus bacteria, as well as the inhibitory effects of ZnO-NWs on this lysis phenomenon. **(A)** The present study examines the effects of exposure to ZnO-NWs on the structure of red blood cells (RBCs). Additionally, the study investigates the impact of S. aureus infection on RBCs. Furthermore, the study explores the combined effects of S. aureus infection and ZnO-NWs exposure on RBCs. The four conditions examined in this study include: normal RBC structure, RBCs exposed to ZnO-NWs, RBCs infected with S. aureus, and RBCs co-cultured with both S. aureus and ZnO-NWs. **(B)** The inhibition of RBC lysis by ZnO-NWs in the presence of S. aureus is investigated in this study **(C)** Additionally, the biocompatibility of ZnO-NWs is evaluated with MTT analysis against HEK-293T.

A hemolysis assay revealed that ZnO-NWs have the potential to reduce hemolysis caused by staphylococcal α-hemolysin, a significant virulence factor of *S. aureus*. Staphylococcal α-hemolysin is known to cause pore formation in the phospholipid bilayers of target cells, leading to cell lysis, and approximately 61% cell lysis was observed in the presence of *S. aureus* alone (**Figure 7A**). However, when exposed to ZnO-NWs, the lysis induced by *S. aureus* was inhibited. At concentrations of 100 μg/ml and 300 μg/ml of ZnO-NWs, less than 21% and 16% (p ≤ 0.001) cell lysis, respectively, was observed. This significant reduction in cell lysis suggests that ZnO-NWs can effectively protect against the damage caused by staphylococcal α-hemolysin, as further elaborated with the SEM images in **Figure 7B**, showing cell damage by *S. aureus* and the co-culture in the presence of ZnO-NWs, showing a reduction in cell lysis.

Interestingly, ZnO-NWs alone, in the absence of *S. aureus*, caused some degree of cell lysis. At concentrations of 100 μg/ml and 300 μg/ml of ZnO-NWs, approximately 9% and 24% cell lysis occurred, respectively. Overall, the hemolysis assay demonstrated that ZnO-NWs can mitigate the hemolysis caused by staphylococcal α-hemolysin and potentially provide protection against its detrimental effects. However, it is essential to consider the potential hemolytic activity of ZnO-NWs at higher concentrations when evaluating their biomedical applications. Further studies are warranted to understand the mechanisms underlying these observations and to optimize the safe and effective use of ZnO-NWs in various biomedical applications.

The results of the MTT assay indicated that the biomimetically synthesized ZnO-NWs did not exhibit any cytotoxic effects on HEK-cells, even at a dose significantly higher than their minimum inhibitory concentration (MIC) of approximately 100 μg ml−1. The experimental findings indicated that the viability of cells decreased as a result of exposure to increasing doses of ZnO-NWs. The findings of this study indicate that ZnO nanoparticles (ZnO-NPs) exhibit favorable characteristics as stable and non-toxic agents, displaying significant antibacterial efficacy without inducing toxicity in host cells (**Figure 7C**).

### Antibacterial potential of ZnO-NWs against skin infection caused by *Saureus*

**Figure 8:**
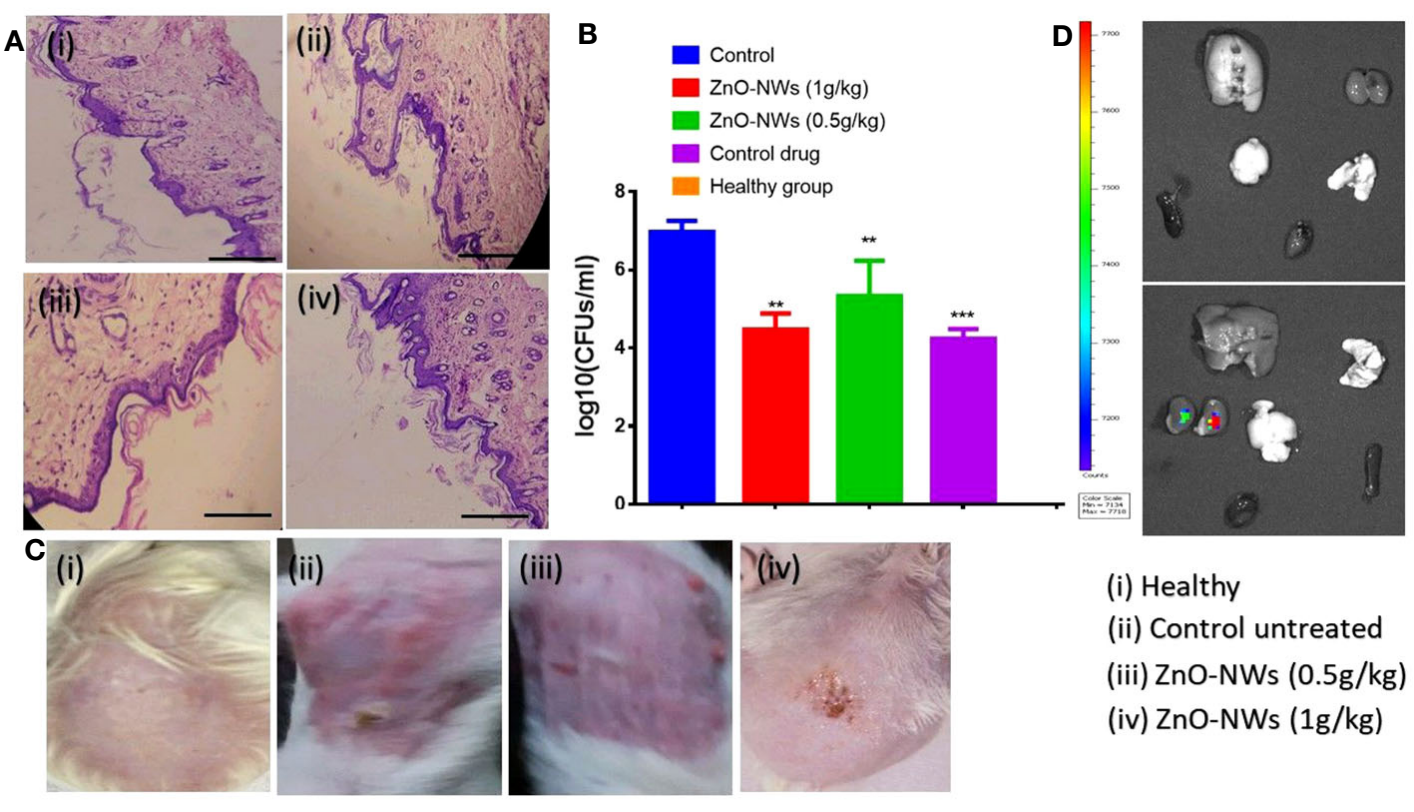
illustrates the potential of ZnO-NWs in the treatment of cutaneous infections in experimental animals. The present study investigates the progression of infection in murine skin models, specifically comparing the effects of PBS treatment, S. aureus infection alone, and S. aureus infection followed by treatment with ZnO-NWs at doses of 1 g kg−1 and 0.5 g kg−1 body weight, respectively. Histopathological investigations of animals subsequent to exposure to ZnO-NWs have been conducted. **(A)** The present study includes photomicrographs of mouse skin depicting four distinct groups: (i) a healthy group, (ii) an untreated control group, (iii) a group treated with 1 g kg−1 body weight of ZnO–NWs, and (iv) a group treated with 0.5 g kg−1 body weight of ZnO–NWs. These groups were established to investigate the effects of ZnO–NPs on the skin of mice. **(B)** The micrographs presented in this study effectively demonstrate that the topical application of ZnO-NWs results in recovery when compared to the untreated control groups. The present study investigates the residual bacterial load present in the skin of experimental animals subsequent to treatment with a formulation containing zinc oxide nanoparticles (ZnO-NWs). **(C)** The experimental procedure involved the topical infection of mice with S. aureus, followed by concurrent treatment with ZnO–NWs (S. aureus + ZnO–NWs). The control group consisted of mice that were inoculated with PBS alone. Following the onset of infection, the skin lesions were excised, homogenized, and subsequently subjected to bacterial enumeration using the colony-forming unit (CFU) assay on the tenth day. **(D)**, the fluorescence *in vivo* imaging method showed that ZnO-NWs were only partially retained in the vital organs. The experiments were conducted in triplicate, and the results are presented as the mean the standard deviation (mean ± SD). The statistical significance was determined using the ***P ≤ 0.001 threshold. ** means p value of 0.01 significant value.

The antibacterial potential of the ZnO-NWs against skin infections caused by *S. aureus* was evaluated both *in vitro* and in mouse models. In the *in vitro* study, ZnO-NWs showed significant antibacterial activity against *S. aureus*. In mouse models, cutaneous microbial infections caused by *S. aureus* were characterized by reddening and disruption of the skin (**Figure 8A**), indicating the severity of the infection. Bacterial load within the infected skin was assessed using the plate count enumeration method. The results revealed that the ZnO-NW treatment led to a remarkable 50% decline in the pathogenic bacterial count compared to the control group as shown in **Figure 8B** (p ≤ 0.005). This significant reduction in the bacterial load demonstrates the potential effectiveness of ZnO-NWs in the treatment of skin infections caused by *S. aureus.*

Overall, the findings from the *in vitro* and *in vivo* studies highlight the strong antibacterial activity of the ZnO-NWs against S. aureus, both in the laboratory setting and in a living organism. This suggests that ZnO-NWs could be promising candidates for the development of therapeutic agents to combat skin infections caused by this pathogen. However, further research and clinical studies are necessary to validate and optimize the use of ZnO-NWs as a potential treatment option for skin infections in humans.

### Histopathology of tissues

Histopathological analysis of tissue samples obtained from mice infected with Staphylococcus aureus and subsequently treated with zinc oxide nanoparticles (ZnO-NWs) yielded significant findings regarding the therapeutic properties of these nanoparticles in the context of cutaneous infections. Within the experimental framework, the control group consisted of individuals who exhibited a state of sound health and were devoid of any infection. Notably, the dermal structure in this group exhibited a typical configuration, characterized by an unimpaired epidermal layer and a negligible or absent inflammatory reaction, as depicted in **Figures 8A, C**.

In contrast, dermal tissues of mice subjected to *S. aureus* infection displayed notable and substantial pathological alterations. The present study observed epidermal layer thinning accompanied by the manifestation of skin disorders and a notable influx of inflammatory cells. Inflammation was also observed in the subcutaneous and connective tissues. Application of zinc oxide nanowhiskers (ZnO-NWs) induces a notable regenerative response within the epidermal layer, leading to a reduction in skin damage. The observed tissues exhibited restoration of their typical structural arrangement accompanied by a notable decrease in inflammation, as evidenced by the diminished presence of inflammatory cells (**Figure 8A**). Furthermore, upon the application of ZnO-NWs in the absence of infection, only minor deviations in the skin were detected, as evidenced by the presence of a limited number of inflammatory cells infiltrating the affected area.

The histopathological analysis revealed that the application of ZnO-NWs had a notable impact on the restoration of dermal tissues afflicted with *Staphylococcus aureus* infection. The observed nanoparticles exhibited notable anti-inflammatory characteristics, resulting in a reduction in tissue damage and facilitation of tissue repair.

To assess the degree of absorption and dispersion of ZnO-NWs after topical administration, mice from each experimental group were administered with Fluorescein Isothiocyanate (FITC)-labeled ZnO-NWs. Subsequent to a 48-hour period, mice were euthanized. Analysis of the outcomes of this research revealed that the kidney and liver demonstrated the most prominent concentration levels of FITC-labeled ZnO-NWs, as illustrated in (**Figure 8D**). Additionally, we conducted assessments on the inherent toxicity of the synthesized ZnO-NWs *in vivo* for their hepatoxicity. The animals that received ZnO-NWs were subjected to analysis of liver function test parameters to assess the toxicity of the nanoparticles *in vivo*. According to the data presented in **Figure SI A**, it can be observed that animals administered with ZnO-NWs had slightly elevated levels of marker enzymes, namely AST and ALT, in comparison to the control animals that did not receive any treatment.

Thus, findings presented in our study confirm that the ZnO-NWs synthesized in-house exhibited no observable toxicity *in vitro*, and the level of toxicity observed *in vivo* was minimal. These results indicate that these nanoparticles can be considered safe for incorporation into medication formulations. This discrepancy implies that such nanoparticles are biocompatible, suggesting their potential for systemic circulation within the organism. The elevated levels of availability in these organs indicate the possibility of ZnO-NWs traversing biological barriers, hinting at their prospective utility in targeted drug delivery systems and other biomedical applications. These findings may lead to advancements in the development of nanotherapeutic agents and contribute to the evolving landscape of nanomedicine.

## Conclusion

In summary, the present investigation has successfully illuminated the methodology for the biosynthesis of Zinc Oxide Nanowhiskers (ZnO-NWs) using chicken egg white as a biological template. The methodological approach necessitated the exposure of the egg white to elevated thermal conditions, with temperatures reaching 180°C. This process resulted in the formation of nanowhiskers exhibiting a diversity in both dimensions and structural configurations. Subsequent to their synthesis, the ZnO-NWs were subjected to meticulous characterization through the application of well-established spectroscopic and microscopic analytical techniques. This rigorous examination corroborated their dimensional properties, validating them within the range of 10-50 nm.

### Top of form

This study aimed to explore the antibacterial and antibiofilm properties of biosynthesized Zinc Oxide Nanowhiskers (ZnO-NWs). The results of this study showed that the proliferation of both susceptible and resistant strains of bacteria was significantly inhibited. The underlying mechanism of the observed antibacterial activity is primarily attributed to the release of Zn^2+^ ions from the ZnO-NWs, leading to the subsequent generation of reactive oxygen species (ROS), which in turn initiates bacterial cell apoptosis.

*In vivo* assessments were performed using murine models to replicate skin infections triggered by *Staphylococcus aureus.* These assessments yield substantiated evidence, underscoring the potential of ZnO-NWs in reducing bacterial proliferation and aiding in the healing process of the infected epidermal tissue. The proficiency of ZnO-NWs in managing cutaneous infections was corroborated through detailed histopathological evaluations and colony-forming unit (CFU) assays, which revealed the biocompatibility of ZnO-NWs, substantiated by the lack of cytotoxic effects on both normal human embryonic kidney cells (HEK-293) and red blood cells (RBCs), even at high concentrations.

The synthesis method introduced in this research is delineated by its uncomplicated nature, economic viability, and eco-friendly approach, paving the way for biomimetic synthesis of nanoparticles from various materials. These research insights underscore the significant potential of ZnO-NWs as a groundbreaking therapeutic modality for managing and alleviating infectious diseases, especially those involving the skin. To fully harness the potential and applications of ZnO NWs in various biomedical fields, further research and developmental efforts are imperative.

## Data availability statement

The raw data supporting the conclusions of this article will be made available by the authors, without undue reservation.

## Ethics statement

Ethical approval was not required for the studies on humans in accordance with the local legislation and institutional requirements because only commercially available established cell lines were used. The animal study was approved by University of Henan animal ethics. The study was conducted in accordance with the local legislation and institutional requirements.

## Author contributions

MR: Conceptualization, Investigation, Methodology, Writing – original draft, Writing – review and editing. SAA: Conceptualization, Investigation, Methodology, Writing – original draft. SK: Conceptualization, Investigation, Methodology, Writing – original draft. JS: Conceptualization, Investigation, Writing – original draft, Writing – review and editing. LA: Data curation, Formal Analysis, Software, Writing – review and editing. NA: Funding acquisition, Resources, Software, Supervision, Validation, Visualization, Writing – review and editing. MS: Formal Analysis, Project administration, Software, Validation, Visualization, Writing – review and editing. SAI: Conceptualization, Data curation, Formal Analysis, Funding acquisition, Methodology, Software, Validation, Writing – review and editing.
