# Corrigendum: Exploring the antibacterial and dermatitis-mitigating properties of chicken egg white-synthesized zinc oxide nano whiskers

**DOI:** 10.3389/fcimb.2024.1376621

**Published:** 2024-02-23

**Authors:** Sidikov Akmal Abdikakharovich, Mohd A. Rauf, Saadullah Khattak, Junaid Ali Shah, Lamya Ahmed Al-Keridis, Nawaf Alshammari, Mohd Saeed, Sadykov Aslan Igorevich

**Affiliations:** ^1^ Department of Dermatology, Ferghana Medical Institute of Public Health, Ferghana, Uzbekistan; ^2^ School of Life Sciences, Henan University, Kaifeng, Henan, China; ^3^ Miller School of Medicine, University of Miami, Miami, FL, United States; ^4^ College of Life Sciences, Jilin University, Changchun, China; ^5^ Biology Department, Faculty of Science, Princess Nourah Bint Abdulrahman University, Riyadh, Saudi Arabia; ^6^ Department of Biology, College of Sciences, University of Hail, Hail, Saudi Arabia

**Keywords:** ZnO-NWs, antibacterial, anti-biofilm, electron microscopy, *E.coli*, *S.aureus*, skin infection

In the published article, there was an error in affiliation(s) of Lamya Ahmed Al-Keridis and Nawaf Alshammari. The authors’ affiliations should be

Lamya Ahmed Al-Keridis ^5^

^5^Biology Department, Faculty of Science, Princess Nourah Bint Abdulrahman University, Riyadh, Saudi Arabia

And Nawaf Alshammari ^6 *^

^6^Department of Biology, College of Sciences, University of Hail, Hail, Saudi Arabia

The authors apologize for this error and state that this does not change the scientific conclusions of the article in any way. The original article has been updated.

