# Supplementary material for: Exploring the antibacterial and dermatitis-mitigating properties of chicken egg white-synthesized zinc oxide nano whiskers
Source: Front Cell Infect Microbiol. 2023 Nov 28;13:1295593. doi: 10.3389/fcimb.2023.1295593 (PMC10719619; doi:10.3389/fcimb.2023.1295593)
Supplement: Supplementary file 1 [file DataSheet_1.docx]

**Characterization of ZnO–NWs employing UV-visible spectroscopy**

The ultraviolet-visible (UV) spectra of zinc oxide nanwhiskers (ZnO-NWs) synthesized using the culture supernatant of S. aureus were measured using a double beam spectrophotometer (Shimadzu) with a resolution of 1 nm.

**Surface and size analysis of  ZnO–NWs employing transmission and scanning electron microscopy**

TEM was used to analyze nanoparticles generated from egg white as bio-template. To make the samples, a drop of reaction result was placed on a gold-coated negative grid and allowed to evaporate. TEM was carried out using a JEOL type electron microscope. The microscope was powered by a 1000 kV accelerating voltage. Scanning electron microscopy SEM was used to examine the surface morphology and size of ZnO-NWs. (JEOL model JSM67500F).

**Determination of Minimum Inhibitory Concentration (MIC)**

Minimum Inhibitory Concentration (MIC) is the lowest concentration of an antimicrobial agent that may prevent the visible growth of microorganisms after stipulated time frame. MIC is considered as a significant parameter in diagnostic laboratories to ascertain sensitivity of a microorganism against a particular antimicrobial agent. The MIC value of *in situ* synthesized ZnO–NWs was determined by microdilution method against several ATCC strains and several clinical isolates *viz. Staphylococcus aureus*, MRSA, *Pseudomonas aeruginosa*, *Escherichia coli* , MREC and Listeria monocytogenes. The values were estimated on the basis of viability test performed in 96-well microdilution plates according to the previously developed protocols.

**Antibacterial potential of *in situ* ZnO–NWs as determined by agar diffusion assay**

The bacterial culture was incubated for an extended period of time in Brain Heart infusion (BHI) medium, followed by centrifugation at 1000G for a duration of 5 minutes. The pellet was subjected to a washing procedure using sterile phosphate-buffered saline (PBS) with a pH of 7.4, followed by resuspension in BHI medium. A 100 μl aliquot of the suspended culture was evenly spread onto the BHI agar plate using a sterile glass spreader. The plate was then incubated at a temperature of 37 °C. The wells were subsequently drilled utilizing a gel borer. Following a 1-hour incubation period, the plate was subjected to escalating concentrations of ZnO-NWs derived from a stock solution with a concentration of 10 mg ml^−1^. The determination of the zone of inhibition was accomplished through the measurement of the extent of bacterial clearance observed after a period of 24 hours. The experimental procedure was conducted in a controlled environment with strict adherence to sterile conditions, employing level-2 bio-safety hoods. The experiments were conducted in triplicate, and subsequently, the mean value of the three replicates was computed. This mean value was then juxtaposed with the reference drug ampicillin, which was employed as the control in the study.

**CFU assessment to evaluate bacterial susceptibility to *in situ* synthesized nanoparticles**

The overnight grown culture of various bacterial isolates were sub-distributed into four culture tubes (adjusted density to 10^6^–10^7^ cells per ml). Further, 100 μl aliquot of ZnO–NWs solution from stock solution of 10 mg ml^−1^ or ampicillin solution (100 μg ml^−1^), negative control (without culture and without formulation) and positive control (culture + 100 μl PBS) was dispensed to corresponding tubes and allowed to incubate for further 4 hours at 37 °C. Thereafter, 100 μL suspension from each of the treated and control group tubes was plated in duplicate up to two different dilutions (1
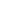
:
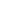
1, 1
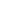
:
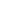
10) on to the TSB agar plates and incubated further at 37 °C. After 24 h of incubation at 37 °C, resultant colony forming units (CFU) at different dilutions were counted, averaged and expressed as log
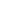
10 CFU ml^−1^ and the counts from two independent experiments were averaged.

**XTT biofilm assay**

The XTT based biofilm assay was performed to assess antibiofilm activity of *in situ* synthesized NWs. Briefly, post mature biofilms formation, the wells of the plate were carefully rinsed with sterile PBS to remove non adherent cells. The mature biofilms were treated with increasing concentration of ZnO–NWs and placed at 37 °C for 48 h. After stipulated incubation period, 2,3-bis(2-methoxy-4-nitro-5-sulfophenyl)-5-[(phenylamino) carbonyl]-2*H*-tetrazolium hydroxide (XTT) solution in PBS, was added at a final concentration of 250 mg ml^−1^. The obtained solution was filter sterilized using a 0.22 mm pore-size filter and stored at −80 °C until required. Menadione solution (0.4 mM) was prepared and filtered immediately just before the commencement of each assay. Adherent cells were washed with PBS (200 μl), followed by addition of XTT, and 2 μl of menadione to each well. The solution was transferred to a new plate after incubation in the dark for 4 h at 37 °C and the colorimetric change in the solution was assessed using a microtitre plate reader (BIORAD Microplate reader at 490 nm). Experiments were performed in triplicate. The data are expressed as means ± SD.

**Hydrophobicity index of nano-particle treated bacterial biofilm**

The overnight grown bacterial cells were resuspended in LB medium and the optical density was adjusted to 1.0 ± 0.01 at a wavelength of 595 nm. 1 ml toluene was added to the cells suspension in a test tube and the tube was vortexed. The biphasic mixture of two phases was allowed to settle for 30 min and the optical density of the aqueous phase was measured. Hydrophobicity index (HI) of microbial cells was determined by using the following equation

| HI% = [(*A*_i_ − *A*_f_)/*A*_i_] × 100 |
| --- |

where *A*_i_ and *A*_f_ are the initial and final optical densities of the aqueous phase. Hydrophobicity of bacterial cells was evaluated by measuring their adherence to organic solvent.

**Bacteria-nanoparticle interaction as revealed by electron microscopy**

Both *E. coli* and *S. aureus* bacteria were exposed to ZnO–NWs for 60 minutes, at 37 °C, with constant agitation (at 250 rpm). The cell suspension was washed 5 times in modified-BHI medium and 3 times in PBS to remove unbound or loosely associated nanoparticles. The cells (approximately 10^8^ CFU) that interacted with ZnO–NPs at different time interval, were prepared and imaged using SEM. Additionally, the bacteria were imaged by Transmission Electron Microscopy (TEM) as well. Briefly, the bacteria, (also approximately 10^9^ CFU) were fixed with 1% glutaraldehyde in PBS and subsequently exposed to 1% osmium tetroxide in water, for 24 hours each. The sample was transferred onto a 0.22 μm pore size filter (Millipore) and substituted with acetone, and subsequently with liquid CO_2_ in a critical point drying apparatus (SPI, USA). Filter paper sections were metalized with gold, by sputter coating, and imaged with a JEOL JSM-6390LV.

**Fluorescence microscopy to determine incurred cell death in ZnO–NWs treated bacterial cells**

Fluorescence microscopy microscope was used to study effect of ZnO–NWs on growing bacterial cells. *S. aureus* and *E. coli* cultures were labelled with propidium iodide (PI) dye as well as syto-9 dye. PI was used to detect of dead cells as PI specifically stains only dead bacteria and gives red fluorescence whereas syto-9 stains only live cells and gives green fluorescence. The ZnO–NWs at their MIC concentrations were added to the overnight grown bacterial cultures and were incubated at 37 °C for 4 h. After the stipulated incubation period, cells were harvested by centrifugation and stained with dyes for 30 min under dark conditions. The cells were centrifuged at 2500*g* for 10 min and the pellet obtained was resuspended in 1 ml of sterile PBS buffer. Cells that were not treated with ZnO–NWs were taken as a control of both bacterial strains. Finally, 10 μl of the above prepared samples were then placed on a glass slide and mounted with the cover slip then cells were examined under a Fluorescence microscopy.

**Acute Toxicity Test for ZnO-NWs**

The study involved the monitoring of hepatic toxicity through the administration of 5 and 10 mg/kg body weight. The aim was to assess the biochemical profiles of serum aspartate aminotransferase (AST) and alanine aminotransferase (ALT) using Standard blood chemistry parameters using a kit from Wuhan Servicebio Technology Co. Ltd. on an automated chemistry analyzer (Chemray 240 Rayto lnc.). Blood cell parameters were analyzed with an automated blood cell analyzer (BC-2800Vet-Mindray Inc.). The 3 group comprised of (i) untreated animals and (ii) mice treated with 5 mg/kg body weight and (iii) mice treated with 10 mg/kg body weight, after a 24-hour period, using retroorbital puncture the blood sample was taken and serum was isolated. The levels of aspartate aminotransferase and alanine aminotransferase were measured in serum according to the guidelines provided by the manufacturer.

**Results**

The Invivo assessments on the inherent toxicity of the synthesized ZnO-NWs was performed. The test particles exhibited a much lower level of hemolysis compared to the control group treated. The animals that received ZnO-NWs were subjected to analysis of liver function test parameters in order to assess the toxicity of the nanoparticles in vivo. According to the data presented in Figure SIA, it can be observed that animals administered with ZnO-NWs had slightly elevated levels of marker enzymes, namely AST and ALT, in comparison to the control animals that did not receive any treatment. The findings presented in this study confirm that the ZnO-NWs synthesized in-house exhibited no observable toxicity in vitro, and the level of toxicity observed in vivo was minimal. These results indicate that these nanoparticles can be considered safe for incorporation into medication formulations**.**

**Figure SIA:** The in vivo toxicity of ZnO-NWs analyzed by liver function test (LFT) for marker enzymes AST and ALT in test (animals treated with different doses of ZnO-NWs) and control (untreated) animals.
